# Genome−wide identification and analysis of *LEA_2* gene family in alfalfa (*Medicago sativa* L.) under aluminum stress

**DOI:** 10.3389/fpls.2022.976160

**Published:** 2022-11-28

**Authors:** Yujing Zhang, Nana Fan, Wuwu Wen, Siyan Liu, Xin Mo, Yuan An, Peng Zhou

**Affiliations:** ^1^ School of Agriculture and Biology, Shanghai Jiao Tong University, Shanghai, China; ^2^ Key Laboratory of Urban Agriculture, Ministry of Agriculture, Shanghai, China

**Keywords:** alfalfa, LEA, gene family, chromosome, gene structure, aluminum stress

## Abstract

Late embryonic development abundant proteins (LEAs) are a large family of proteins commonly existing in plants. LEA_2 is the largest subfamily in the LEA, it plays an important role in plant resistance to abiotic stress. In order to explore the characteristics of *LEA_2* gene family members in alfalfa (*Medicago sativa* L.), 155 members of *LEA_2* (*MsLEA_2*) family were identified from alfalfa genome. Bioinformatics analysis was conducted from the aspects of phylogenetic relationship, chromosome distribution, chromosome colinearity, physical and chemical properties, motif composition, exon-intron structure, *cis*-element and so on. Expression profiles of *MsLEA_2* gene were obtained based on Real-time fluorescent quantitative PCR (qRT-PCR) analysis and previous RNA-seq data under aluminum (Al) stress. Bioinformatics results were shown that the *MsLEA_2* genes are distributed on all 32 chromosomes. Among them, 85 genes were present in the gene clusters, accounting for 54.83%, and chromosome Chr7.3 carries the largest number of *MsLEA_2* (19 *LEA_2* genes on Chr7.3). Chr7.3 has a unique structure of *MsLEA_2* distribution, which reveals a possible special role of Chr7.3 in ensuring the function of *MsLEA_2*. Transcriptional structure analysis revealed that the number of exons in each gene varies from 1 to 3, and introns varies from 0 to 2. *Cis*-element analysis identified that the promoter region of *MsLEA_2* is rich in ABRE, MBS, LTR, and MeJARE, indicating *MsLEA_2* has stress resistance potential under abiotic stress. RNA-seq data and qRT-PCR analyses showed that most of the *MsLEA_2* members were up-regulated when alfalfa exposed to Al stress. This study revealed that phylogenetic relationship and possible function of *LEA_ 2* gene in alfalfa, which were helpful for the functional analysis of LEA_ 2 proteins in the future and provided a new theoretical basis for improving Al tolerance of alfalfa.

## Introduction

Late Embryogenesis Abundant Protein (LEA) is a big protein family ubiquitous in plants. They are abundantly expressed in plants under various abiotic stresses such as low temperature, drought, and so on, to enhance the plants’ resistance. Therefore, LEA is an important resistance protein in plants. Since Dure et al. discovered LEA in cotton cotyledons in 1981 (Dure et al., 1981), LEA protein has been subsequently founded in many other plants such as *Oryza sativa*, *Zea mays*, and *Hordeum vulgare* etc (Espelund et al., 1995; Li and Cao, 2015; Xue et al., 2021).

Most LEA proteins are small proteins with molecular weight from 10 × 10^3^ to 30 × 10^3^ Daltons. Their common feature in sequence is that they contain more polar amino acids residues such as glycine, alanine, serine and threonine than ordinary proteins. The biggest feature of the secondary structure is that the content of random coils is high and the sequence is highly disordered, which belongs to intrinsically disordered proteins (IDPs) (Chakrabortee et al., 2010). These characteristics make this type of protein extremely hydrophilic, thermally stable (Browne et al., 2004; Kovacs and Tompa, 2012), and its conformation is highly plastic when interacting with other biomolecules (Kovacs et al., 2013).

According to the amino acid sequence of 8 conserved PFAM domains, LEA proteins were classified into 9 families (LEA_1-LEA_6, Dehydrin, SMP and AtM) (Finn et al., 2014). The LEA_2 family is obviously different from other LEA proteins. Their secondary structure contains less random coils than other LEA proteins and is rich in β-sheets. The protein sequence of many LEA_2 contains one or several copies of the water stress and hypersensitive response domain (WHy). This domain is not very conserved, with about 100 amino acids residues. The core sequence of WHy domain often contains three amino acid residues of NPN/Y (Kovacs et al., 2013). Until now, the function of WHy domain is still not well understood. It is reported that WHy domain may have the function of protecting protein from denaturation (Anderson et al., 2015; Jiang et al., 2017). LEA_2 can improve plant stress tolerance, which may be related to the WHy domain.

Under abiotic stress, the way plants evolve to adapt to the environment is gene family expansion through tandem repeats. As the largest *LEA* family, *LEA_2* has a large number of members (Artur et al., 2019), but in general, the motifs of *LEA_2* has the same phylogeny with motifs of other subfamilies in LEA family. During the continuous expansion of the *LEA_2* family in the form of tandem repeats, the *LEA_2* family has also produced various functions. This explains the reason why the functions of the members of *LEA_2s* are diverse under stresses (Artur et al., 2019).

Alfalfa (*Medicago sativa* L.) is a perennial leguminous herb with good palatability and can be used in multiple crops within a year, and is widely grown worldwide (He et al., 2020). Alfalfa, as the primary feed for livestock, is an important foundation for the prosperity of the dairy industry. But it is sensitive to aluminum (Al) stress (Rechcigl et al., 1988). Using modern biological technology to study alfalfa stress genes, and using molecular breeding technology to cultivate new varieties, are important ways to increase alfalfa planting area, increase alfalfa yield, and support the development of the dairy industry (Hrbackova et al., 2020). As *LEA_2* gene family has been assumed to play a major role in plants abiotic stress resistance, it is necessary to study *LEA_2* in alfalfa. In this study, a total of 155 members of the alfalfa *LEA_2* family (*MsLEA_2*) were identified. And bioinformatics was used to analyze the composition, domain, and *cis*-acting elements of *MsLEA_2* family. The expression of *MsLEA_2s* under Al stress at different time points was analyzed by using the previous transcriptome data in our laboratory and verified by Real-time fluorescent quantitative PCR, thereby further elucidating the Al tolerance mechanism of alfalfa and providing candidate genes for Al tolerance breeding of alfalfa.

## Materials and methods

### Identification and analysis of LEA_2 family members in alfalfa

The genome-wide data of *Arabidopsis thaliana* were downloaded from the TAIR website (https://www.arabidopsis.org/) (Lamesch et al., 2012). The genome-wide data of the cultivated alfalfa (cultivar XinJiangDaYe) are obtained in the attachment of the paper (Chen et al., 2020b). In this paper, members of the LEA_2 family were determined according to the classification method of Hundert mark (2008) (Hundertmark and Hincha, 2008).

Employed all the four Arabidopsis *AtLEA_2* gene family members (*AtLEA14*, *AtLEA26*, *AtLEA27*, *NHL26*) reported in the literature as reference sequences (Françoise et al., 2013), bidirectional BLAST alignment was performed using TBtools (set E<1×10^-5^) (Chen et al., 2020a).

The PFAM number (PF03168) of the LEA_2 was retrieved from the Pfam database website (http://Pfam.xfam.org) (Finn et al., 2014); the LEA_2 proteins sequences were extracted from the alfalfa genome using the software HMMER v3.3.1 (set E<1 ×10^-5^) (Finn et al., 2011). After removing redundancy, the ID information of 273 possible *MsLEA_2* gene families were obtained. After the candidate sequences obtained by the two methods were combined and deduplicated, the sequences obtained by screening were further identified by NCBI-CDD and SMART database, and a total of 155 possible members of the *MsLEA_2* family were obtained.

### Multiple sequence alignment and phylogenetic analysis

The amino acid sequences of 155 MsLEA_2 and 4 AtLEA_2 were extracted, and Clustal X tool was used to perform multiple alignment analysis in MEGA v10.2.6 (Kumar et al., 2018) with default parameters. The analysis results were used MEGA v10.2.6 analysis software to construct a phylogenetic tree using the maximum likelihood (ML) method, and the bootstrap test value was set to 1000.

### Synteny analysis and chromosomal distribution of *MsLEA_2* genes

Synteny analysis was performed using MCScanX implementing the default parameters, the output was then transformed to visual result by TBtools, and the members of the *MsLEA_2* family are marked with names (Wang et al., 2013). Using TBtools to extract the location information of all *MsLEA_2* genes in the alfalfa genome, the online tool MapGene2Chrom (http://mg2c.iask.in/mg2c_v2.0/) was used to map the chromosomal location of members of the *MsLEA_2* family (Chao et al., 2015).

### Physicochemical properties and subcellular localization of protein

Molecular weight (MW), isoelectric point (PI) and grand average of hydropathicity (GRAVY) were analyzed using the ExPaSy protein server (https://web.expasy.org/translate/). Prediction of subcellular localization of LEA_2 protein in alfalfa by CELLO (http://cello.life.nctu.edu.tw/), an online website. (Yu et al., 2004; Yu et al., 2006)

### Gene structure, motif composition and *cis*-elements analysis

Gene structure was analyzed using the online tool Gene Structure Displays (http://gsds.gao-lab.org/). The Motif distribution of MsLEA_2 protein was analyzed by MEME (https://meme-suite.org/meme/tools/meme). Due to the large differences between the sequences of the *MsLEA_2* family, the maximum number of motifs was set to 10, the minimum base width was 6, and the maximum base sequence width was 50.

The 2000bp sequence upstream of the initiation codon of the *MsLEA_2* was intercepted from the alfalfa genome data as the promoter region, and the promoter elements were analyzed using the plant *cis*-element database PlantCARE (http://bioinformatics.psb.ugent.be/webtools/plantcare/html/) to complete (Lescot et al., 2002). The *cis*-elements distribution on *MsLEA_2* promoters were visualized using TBtools.

### Gene ontology annotations

Gene sets were constructed using members of the *MsLEA_2* gene family, and GO annotation analysis was performed using the online GOEAST database (http://omicslab.genetics.ac.cn/GOEAST/index.php) (Zheng and Wang, 2008).

### Plant materials, growth conditions and treatments

Gently wash off the coating of alfalfa seeds (WL-525HQ genotype, from the Chinese National Seed Group Corporation) with clean water, select seeds with a uniform and plump shape and no obvious pests and diseases, and evenly scatter them on a metal tray covered with double-layer filter paper to ensure that the spacing between each seed is consistent, and the tray is sealed with plastic wrap to ensure that the moisture in the tray is appropriate. The growth camber conditions were: 16-h photoperiod, 25°C/20°C (day/night), relative humidity of 60–65%, and light intensity of 400 μmol m2 s^-1^. After cultivating for one week, select 5 uniform seedlings, wrap the stems with sponge and fix them on the foam floating plate (12 holes very plate), and transplant them to the foam board floating on the 1/2 Hoagland nutrient solution (pH5.8). During the cultivation period, the 1/2 Hoagland nutrient solution was changed every 2 days, all seedlings were grown in the growth camber with the growth conditions mentioned above.

For aluminum (Al) treatments, 14-days-old seedlings were treated with Al treatments in the ½-strength Hoagland’s nutrient solution (pH 4.5) containing AlCl_3_ (100μM). The whole plant was collected at 0, 3, 6, 12 and 24 hours after treatment, washed with deionized water, lightly dipped in water with absorbent paper, wrapped in tinfoil and quickly frozen in liquid nitrogen, and stored at -80°C.

### Expression profiling of the *MsLEA_2*

The expression profiles of 36 *MsLEA_2* genes were extracted from previous RNA-seq of our laboratory. RNA-seq data is stored on the Biomarker cloud platform (Biomarker Technologies, Beijing, China) (https://international.biocloud.net/zh/dashboard).

For Real-time fluorescent quantitative PCR (qRT-PCR), total RNA was extracted by TransZol Up Plus RNA Kit (Transgen, China). The first-strand cDNA was synthesized by a TransScript One-Step gDNA Removal and cDNA Synthesis SuperMix (Transgen, China). The methods of qRT-PCR were mentioned in Cheng et al., 2020. Triplicate quantitative assays of six gene (*MsLEA_2-6*, *MsLEA_2-45*, *MsLEA_2-51*, *MsLEA_2-82*, *MsLEA_2-120*, *MsLEA_2-154*) was performed using the TOP Green Supermix (TransGen) on a Bio-Rad CFX connect system. The MsEF-α gene was used as an endogenous control. All the primers used in qRT-PCR were shown in **Supplementary Table S1**. The relative expression value was determined using the 2 ^-△△CT^ method (Livak and Schmittgen, 2001). Three biological replicates were examined.

## Results

### Identification of *LEA_2* gene family members in alfalfa

A total of 155 members of the *MsLEA_2* family were obtained. Except for one named as *MsLEA2* in the previous work, the remaining 154 genes were named according to the order of the genes on the chromosome, and the names were simplified as *MsLEA_2-1*, *MsLEA_2-2*…*MsLEA_2-154* (All the sequence ID and related gene name was shown in **Table 1**).

**Table 1:** Prediction characteristic information table of *MsLEA _ 2* gene family proteins.

Seq.ID	Gene name	Chr.ID	Number of bases	Theoretical pI	MW (kD)	GRAVY	Number of aa	Predicted localization^a^
MS.gene034785.t1	*MsLEA_2-1*	Chr1.1	629	9.51	24090.74	-0.09	209	plas
MS.gene034786.t1	*MsLEA_2-2*	Chr1.1	629	9.6	24191.17	-0.137	209	extra
MS.gene034789.t1	*MsLEA_2-3*	Chr1.1	743	8.87	28486.09	-0.247	247	plas
MS.gene034792.t1	*MsLEA_2-4*	Chr1.1	666	10.11	25391.17	0.056	212	plas
MS.gene062187.t1	*MsLEA_2-5*	Chr1.1	752	9.93	27943.02	-0.097	250	mito
MS.gene49981.t1	*MsLEA_2-6*	Chr1.1	695	9.59	25938.69	-0.029	231	plas
MS.gene07483.t1	*MsLEA_2-7*	Chr1.2	752	9.93	27880.95	-0.217	250	mito
MS.gene93831.t1	*MsLEA_2-8*	Chr1.2	695	9.59	25924.66	-0.263	231	plas
MS.gene41065.t1	*MsLEA_2-9*	Chr1.3	629	9.49	24134.8	-0.103	209	plas
MS.gene41066.t1	*MsLEA_2-10*	Chr1.3	629	9.3	24099.02	-0.057	209	plas
MS.gene45444.t1	*MsLEA_2-11*	Chr1.3	629	9.44	24128.99	-0.141	209	extra
MS.gene45443.t1	*MsLEA_2-12*	Chr1.3	629	9.49	24134.8	-0.102	209	plas
MS.gene41069.t1	*MsLEA_2-13*	Chr1.3	746	8.87	28603.15	-0.168	248	plas
MS.gene91390.t1	*MsLEA_2-14*	Chr1.3	695	9.59	25964.77	-0.234	231	plas
MS.gene055662.t1	*MsLEA_2-15*	Chr1.4	629	9.49	24134.8	-0.102	209	plas
MS.gene055661.t1	*MsLEA_2-16*	Chr1.4	629	9.3	24099.02	-0.21	209	plas
MS.gene055658.t1	*MsLEA_2-17*	Chr1.4	746	8.87	28586.17	-0.16	248	plas
MS.gene055657.t1	*MsLEA_2-18*	Chr1.4	758	8.85	29077.07	0.127	252	plas
MS.gene004955.t1	*MsLEA_2-19*	Chr1.4	752	10.1	27966.1	-0.115	250	mito
MS.gene99672.t1	*MsLEA_2-20*	Chr1.4	695	9.59	25924.66	-0.263	231	plas
MS.gene057020.t1	*MsLEA_2-21*	Chr2.1	791	10.34	29033.84	-0.161	263	nuc
MS.gene034413.t1	*MsLEA_2-22*	Chr2.1	644	9.41	23895.69	-0.041	214	plas
MS.gene034415.t1	*MsLEA_2-23*	Chr2.1	698	9.81	25676.09	-0.136	232	plas
MS.gene034417.t1	*MsLEA_2-24*	Chr2.1	665	9.22	24704.58	0.124	221	plas
MS.gene35450.t1	*MsLEA_2-25*	Chr2.1	644	9.41	23895.69	-0.041	214	plas
MS.gene35448.t1	*MsLEA_2-26*	Chr2.1	698	9.81	25676.09	-0.136	232	plas
MS.gene35446.t1	*MsLEA_2-27*	Chr2.1	665	9.34	24723.62	0.118	221	plas
MS.gene047524.t1	*MsLEA_2-28*	Chr2.1	797	9.26	30356.36	-0.301	265	nuc
MS.gene02286.t1	*MsLEA_2-29*	Chr2.1	608	9.63	22952.66	-0.071	202	extra
MS.gene051940.t1	*MsLEA_2-30*	Chr2.2	791	10.4	29032.85	-0.206	263	nuc
MS.gene84289.t1	*MsLEA_2-31*	Chr2.2	644	9.41	23881.66	-0.039	214	plas
MS.gene84290.t1	*MsLEA_2-32*	Chr2.2	698	9.81	25685.1	-0.135	232	plas
MS.gene84293.t1	*MsLEA_2-33*	Chr2.2	665	9.38	24721.61	0.132	221	plas
MS.gene97049.t1	*MsLEA_2-34*	Chr2.2	437	8.4	16361.83	-0.132	145	nuc
MS.gene01365.t1	*MsLEA_2-35*	Chr2.2	797	9.35	30268.25	-0.293	265	nuc
MS.gene02171.t1	*MsLEA_2-36*	Chr2.2	554	9.49	21072.55	0.066	184	plas
MS.gene02173.t1	*MsLEA_2-37*	Chr2.2	554	9.49	21072.55	0.066	184	plas
MS.gene01316.t1	*MsLEA_2-38*	Chr2.2	608	9.63	22952.66	-0.071	202	extra
MS.gene76140.t1	*MsLEA_2-39*	Chr2.3	791	10.4	29133.92	-0.25	263	nuc
MS.gene01775.t1	*MsLEA_2-40*	Chr2.3	797	9.35	30280.3	0.137	265	nuc
MS.gene002335.t1	*MsLEA_2-41*	Chr2.3	608	9.63	22952.66	-0.071	202	extra
MS.gene85211.t1	*MsLEA_2-42*	Chr2.4	791	10.46	29091.92	0.083	263	nuc
MS.gene002388.t1	*MsLEA_2-43*	Chr2.4	797	9.34	30296.3	-0.313	265	nuc
MS.gene01727.t1	*MsLEA_2-44*	Chr2.4	608	9.63	22952.66	-0.254	202	extra
MS.gene008386.t1	*MsLEA_2-45*	Chr3.1	758	10.09	28672.38	-0.101	252	nuc
MS.gene70572.t1	*MsLEA_2-46*	Chr3.1	749	9.18	27653.06	-0.082	249	plas
MS.gene70571.t1	*MsLEA_2-47*	Chr3.1	617	8.56	23018.56	-0.071	205	extra
MS.gene70570.t1	*MsLEA_2-48*	Chr3.1	641	9.08	24235.54	0.098	213	plas
MS.gene70569.t1	*MsLEA_2-49*	Chr3.1	638	8.59	24064.12	0.072	212	extra
MS.gene06817.t1	*MsLEA_2-50*	Chr3.1	635	9.66	23681.47	0	211	plas
MS.gene32816.t1	*MsLEA_2-51*	Chr3.1	782	9.94	28749.18	-0.24	260	nuc
MS.gene019021.t1	*MsLEA_2-52*	Chr3.2	935	4.83	34250.28	-0.283	311	cytop
MS.gene049634.t1	*MsLEA_2-53*	Chr3.2	758	10.13	28629.32	-0.344	252	nuc
MS.gene25896.t1	*MsLEA_2-54*	Chr3.2	749	9.18	27653.06	-0.095	249	plas
MS.gene25895.t1	*MsLEA_2-55*	Chr3.2	617	8.56	22986.5	-0.01	205	extra
MS.gene25894.t1	*MsLEA_2-56*	Chr3.2	641	9.08	24235.54	0.098	213	plas
MS.gene25893.t1	*MsLEA_2-57*	Chr3.2	638	8.59	24064.12	0.072	212	extra
MS.gene057250.t1	*MsLEA_2-58*	Chr3.2	635	9.66	23681.47	0	211	plas
MS.gene015019.t1	*MsLEA_2-59*	Chr3.2	782	9.94	28749.18	-0.24	260	nuc
MS.gene29410.t1	*MsLEA_2-60*	Chr3.3	935	4.79	34216.16	-0.313	311	cytop
MS.gene89438.t1	*MsLEA_2-61*	Chr3.3	1923	4.75	34232.12	-0.338	311	cytop
MS.gene55676.t1	*MsLEA_2-62*	Chr3.3	758	10.13	28602.34	-0.308	252	nuc
MS.gene022243.t1	*MsLEA_2-63*	Chr3.3	749	9.18	27653.06	-0.082	249	plas
MS.gene022242.t1	*MsLEA_2-64*	Chr3.3	617	8.56	22972.47	-0.01	205	extra
MS.gene022241.t1	*MsLEA_2-65*	Chr3.3	641	9.08	24270.63	-0.478	213	plas
MS.gene022239.t1	*MsLEA_2-66*	Chr3.3	1903	8.8	26435.1	0.284	235	extra
MS.gene044220.t1	*MsLEA_2-67*	Chr3.3	617	8.95	22907.44	0.006	205	extra
MS.gene06604.t1	*MsLEA_2-68*	Chr3.3	635	9.66	23681.47	0	211	plas
MS.gene012812.t1	*MsLEA_2-69*	Chr3.3	782	10	28787.28	0.137	260	extra
MS.gene03678.t1	*MsLEA_2-70*	Chr3.4	932	4.82	34163.1	-0.32	310	cytop
MS.gene05079.t1	*MsLEA_2-71*	Chr3.4	758	10	28653.29	-0.32	252	nuc
MS.gene074408.t1	*MsLEA_2-72*	Chr3.4	749	9.18	27653.06	-0.247	249	plas
MS.gene074409.t1	*MsLEA_2-73*	Chr3.4	617	8.56	23032.57	0.008	205	extra
MS.gene074411.t1	*MsLEA_2-74*	Chr3.4	638	8.59	24032.06	-0.057	212	extra
MS.gene013497.t1	*MsLEA_2-75*	Chr3.4	635	9.66	23681.47	-0.206	211	plas
MS.gene37424.t1	*MsLEA_2-76*	Chr3.4	782	10	28787.28	-0.273	260	extra
MS.gene051327.t1	*MsLEA_2-77*	Chr4.1	659	9.47	24725.6	-0.05	219	plas
MS.gene044760.t1	*MsLEA_2-78*	Chr4.1	665	9.57	24891.84	0.081	221	plas
MS.gene028407.t1	*MsLEA_2-79*	Chr4.2	659	9.55	24698.53	-0.071	219	plas
MS.gene98613.t1	*MsLEA_2-80*	Chr4.2	656	9.54	24567.38	-0.008	218	plas
MS.gene70399.t1	*MsLEA_2-81*	Chr4.3	659	9.55	24698.53	-0.071	219	plas
MS.gene66696.t1	*MsLEA_2-82*	Chr4.3	659	9.57	24761.66	0.025	219	plas
MS.gene35049.t1	*MsLEA_2-83*	Chr4.4	659	9.47	24699.52	-0.071	219	plas
MS.gene88169.t1	*MsLEA_2-84*	Chr4.4	665	9.57	24891.84	0.081	221	plas
MS.gene044751.t1	*MsLEA_2-85*	Chr4.4	635	7.75	23556.3	0.123	211	plas
MS.gene017274.t1	*MsLEA_2-86*	Chr5.1	776	10.03	29026.85	-0.327	258	nuc
MS.gene99388.t1	*MsLEA_2-87*	Chr5.1	788	9.74	29619	-0.163	262	nuc
MS.gene016934.t1	*MsLEA_2-88*	Chr5.1	1482	4.79	36243.27	-0.478	323	cytop
MS.gene99096.t1	*MsLEA_2-89*	Chr5.2	776	10.1	29107.92	-0.297	258	nuc
MS.gene03042.t1	*MsLEA_2-90*	Chr5.2	788	9.83	29673.03	-0.195	262	nuc
MS.gene016859.t1	*MsLEA_2-91*	Chr5.2	788	9.83	29673.03	-0.195	262	nuc
MS.gene016861.t1	*MsLEA_2-92*	Chr5.2	785	9.87	29386.65	0.049	261	nuc
MS.gene009616.t1	*MsLEA_2-93*	Chr5.3	776	10.1	29107.92	-0.102	258	nuc
MS.gene37831.t1	*MsLEA_2-94*	Chr5.3	788	9.83	29649.05	-0.168	262	plas
MS.gene37827.t1	*MsLEA_2-95*	Chr5.3	791	9.84	29684.25	-0.126	263	plas
MS.gene70835.t1	*MsLEA_2-96*	Chr5.3	1482	4.79	36300.32	-0.478	324	cytop
MS.gene26221.t1	*MsLEA_2-97*	Chr5.4	776	10.1	29073.91	-0.29	258	nuc
MS.gene39820.t1	*MsLEA_2-98*	Chr5.4	788	9.91	29676.16	-0.297	262	plas
MS.gene39818.t1	*MsLEA_2-99*	Chr5.4	2030	9.92	32466.11	-0.244	287	plas
MS.gene016932.t1	*MsLEA_2-100*	Chr5.4	1479	4.79	36243.27	-0.161	323	cytop
MS.gene042891.t1	*MsLEA_2-101*	Chr6.1	650	9.26	25041.44	-0.177	216	plas
MS.gene042892.t1	*MsLEA_2-102*	Chr6.1	578	9.51	21958.67	-0.332	192	plas
MS.gene79803.t1	*MsLEA_2-103*	Chr6.2	632	9.58	24163.81	-0.078	210	plas
MS.gene93992.t1	*MsLEA_2-104*	Chr6.2	653	9.28	25230.61	-0.188	217	plas
MS.gene93991.t1	*MsLEA_2-105*	Chr6.2	644	9.2	24917.31	-0.144	214	plas
MS.gene81842.t1	*MsLEA_2-106*	Chr6.3	650	9.46	24941.36	-0.18	216	plas
MS.gene81838.t1	*MsLEA_2-107*	Chr6.3	552	9.36	21733.02	-0.074	184	plas
MS.gene81837.t1	*MsLEA_2-108*	Chr6.3	644	9.39	24804.23	-0.124	214	plas
MS.gene72628.t1	*MsLEA_2-109*	Chr6.4	644	9.37	24908.29	-0.171	214	plas
MS.gene025855.t1	*MsLEA_2-110*	Chr7.1	674	9.84	24559.76	0.191	224	plas
MS.gene025648.t1	*MsLEA_2-111*	Chr7.1	632	9.8	23827.55	0.036	210	plas
MS.gene025647.t1	*MsLEA_2-112*	Chr7.1	695	9.58	26516.78	-0.188	231	extra
MS.gene025646.t1	*MsLEA_2-113*	Chr7.1	683	9.57	26335.6	-0.15	227	plas
MS.gene023982.t1	*MsLEA_2-114*	Chr7.1	1986	8.67	13437.39	-0.355	121	nuc
MS.gene024387.t1	*MsLEA_2-115*	Chr7.2	674	9.76	24531.7	-0.082	224	plas
MS.gene85469.t1	*MsLEA_2-116*	Chr7.2	3036	4.42	16574.91	0.032	152	cytop
MS.gene85468.t1	*MsLEA_2-117*	Chr7.2	1905	8.67	13405.33	-0.335	121	nuc
MS.gene22751.t1	*MsLEA_2-118*	Chr7.3	674	9.76	24531.7	0.196	224	plas
MS.gene26475.t1	*MsLEA_2-119*	Chr7.3	632	9.8	23827.55	0.036	210	plas
MS.gene007136.t1	*MsLEA_2-120*	Chr7.3	632	9.8	23827.55	0.036	210	plas
MS.gene007138.t1	*MsLEA_2-121*	Chr7.3	683	9.57	26305.51	-0.021	227	plas
MS.gene007140.t1	*MsLEA_2-122*	Chr7.3	683	9.57	26305.51	-0.161	227	plas
MS.gene051893.t1	*MsLEA_2-123*	Chr7.3	683	9.57	26335.6	-0.15	227	plas
MS.gene051892.t1	*MsLEA_2-124*	Chr7.3	695	9.58	26516.78	-0.095	231	extra
MS.gene051891.t1	*MsLEA_2-125*	Chr7.3	664	9.71	25613.77	-0.148	221	plas
MS.gene051890.t1	*MsLEA_2-126*	Chr7.3	695	9.58	26516.78	-0.095	231	extra
MS.gene025644.t1	*MsLEA_2-127*	Chr7.3	683	9.57	26335.6	-0.15	227	plas
MS.gene025643.t1	*MsLEA_2-128*	Chr7.3	692	9.47	26738.78	-0.32	230	extra
MS.gene025641.t1	*MsLEA_2-129*	Chr7.3	419	9.2	16181.64	-0.286	139	mito
MS.gene050363.t1	*MsLEA_2-130*	Chr7.3	686	9.39	25870.93	-0.21	228	plas
MS.gene025640.t1	*MsLEA_2-131*	Chr7.3	686	9.39	25870.93	-0.21	228	plas
MS.gene007142.t1	*MsLEA_2-132*	Chr7.3	692	9.4	26719.73	-0.314	230	extra
MS.gene007145.t1	*MsLEA_2-133*	Chr7.3	692	9.29	26751.02	-0.153	230	plas
MS.gene007146.t1	*MsLEA_2-134*	Chr7.3	686	9.39	25870.93	-0.282	228	plas
MS.gene51223.t1	*MsLEA_2-135*	Chr7.3	3017	5.14	18077.01	0.148	163	plas
MS.gene51224.t1	*MsLEA2*	Chr7.3	2038	4.6	16745.27	0.233	152	cytop
MS.gene050698.t1	*MsLEA_2-136*	Chr7.4	674	9.76	24531.7	0.196	224	plas
MS.gene058679.t1	*MsLEA_2-137*	Chr7.4	3029	4.53	15810.07	-0.07	144	cytop
MS.gene036506.t1	*MsLEA_2-138*	Chr8.1	746	9.6	28064.57	0.02	248	plas
MS.gene58402.t1	*MsLEA_2-139*	Chr8.1	746	9.6	28064.57	-0.029	248	plas
MS.gene82947.t1	*MsLEA_2-140*	Chr8.1	611	9.86	22627.33	-0.082	203	plas
MS.gene019702.t1	*MsLEA_2-141*	Chr8.1	793	5.12	16733.33	0.196	153	cytop
MS.gene060478.t1	*MsLEA_2-142*	Chr8.1	809	8.97	31109.05	-0.403	269	plas
MS.gene041542.t1	*MsLEA_2-143*	Chr8.2	740	9.6	27745.19	-0.02	246	plas
MS.gene056563.t1	*MsLEA_2-144*	Chr8.2	611	9.86	22627.33	0.137	203	plas
MS.gene79837.t1	*MsLEA_2-145*	Chr8.2	792	5.12	16705.3	-0.026	153	cytop
MS.gene82004.t1	*MsLEA_2-146*	Chr8.2	857	8.89	33266.37	-0.559	285	nuc
MS.gene035313.t1	*MsLEA_2-147*	Chr8.3	740	9.68	27804.26	-0.031	246	plas
MS.gene067988.t1	*MsLEA_2-148*	Chr8.3	611	9.86	22627.33	-0.146	203	plas
MS.gene92109.t1	*MsLEA_2-149*	Chr8.3	831	5.12	16671.24	-0.083	153	cytop
MS.gene08516.t1	*MsLEA_2-150*	Chr8.3	851	8.96	32946.05	-0.522	283	nuc
MS.gene036507.t1	*MsLEA_2-151*	Chr8.4	746	9.6	28064.57	-0.029	248	plas
MS.gene83400.t1	*MsLEA_2-152*	Chr8.4	611	9.86	22627.33	0.137	203	plas
MS.gene79691.t1	*MsLEA_2-153*	Chr8.4	838	5.12	16733.33	-0.105	153	cytop
MS.gene067336.t1	*MsLEA_2-154*	Chr8.4	851	8.96	32946.05	-0.522	283	nuc
**average**	**-**	**-**	**901**	**8.83**	**25426.44**	**-0.130**	**223**	**-**

a Predicted subcellular localization was analyzed by CELLO Web server (http://cello.life.nctu.edu.tw) (Yu et al., 2004; Yu et al., 2006). Abbreviations were used to indicate the subcellular localization of LEA_2 proteins: “cytop” refer to Cytoplasmic; “extra” refer to Extracellular; “mito” refer to Mitochondrial; “nuc” refer to Nuclear; “plas” refer to Plasma Membrane.

### Evolution and interspecific collinearity analysis of *MsLEA_2* genes

Using the Clustal X program, 4 *AtLEA_2s* and 155 *MsLEA_2s* were subjected to multiple sequence alignment analysis. The alignment results were processed using the program MEGA v10.2.6 (http://megasoftware.net), and the phylogenetic tree of the *MsLEA_2* gene was generated by the maximum likelihood (ML) method, and the bootstrap value was set to 1000. Due to differences in gene naming in different literatures, to avoid confusion, **Table 2** lists the location, gene name and proven molecular functions of the members of the *AtLEA_2*, including *AtLEA14* (At1g01470) (Li et al., 2014), *AtLEA26* (At2g44060) (Kamil et al., 2011), *AtLEA27* (At2g46140) (Dang et al., 2014), *AtNHL26* (At5g53730) (Françoise et al., 2013).

**Table 2:** Correspondence between different nomenclatures of *AtlLEA_2* Gene Family.

Locus tag	Gene name	PFAM NO.	SubcellμLar localization	MolecμLar function
At1g01470	*AtLEA14*	PF03168	cytosol	Improve plant resistance to drought/salt stress (Li et al., 2014) (Jia et al., 2014)
At2g44060	*AtLEA26*	PF03168	cytosol, plasma membrane	Improve plant resistance to drought/cadmium stress (Kamil et al., 2011)
At2g46140	*AtLEA27 AtLEA2R*	PF03168	cytosol, plasma membrane	Protects yeast cells during freeze/drying (Dang et al., 2014)
At5g53730	*AtNHL26*	PF03168	plasmodesmata, endoplasmic reticulum	Affects plasmodesmata permeability or sugar signaling (Françoise et al., 2013)

**Figure 1:**
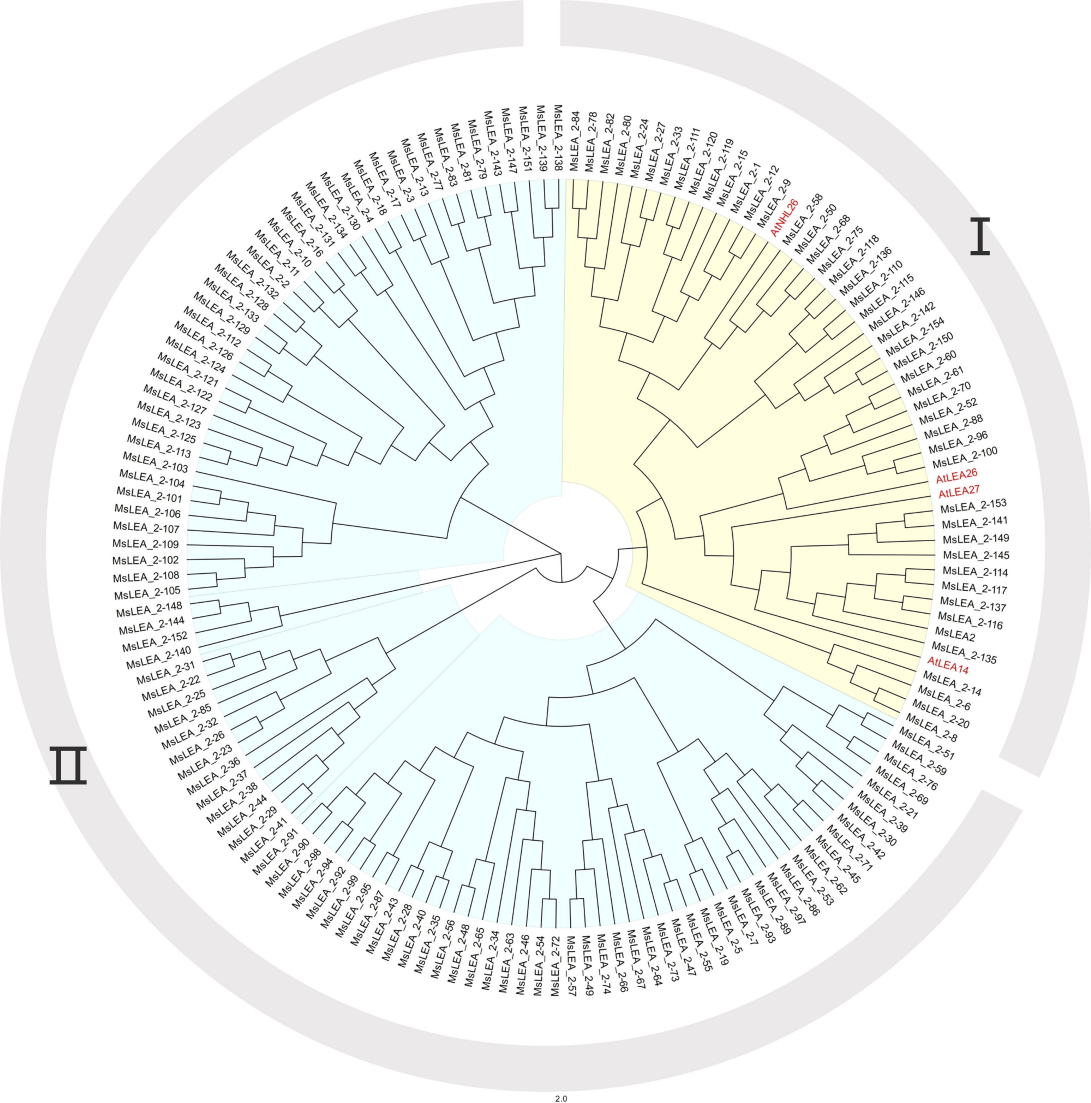
Phylogenetic analysis of LEA_2 protein family from *Medicago sativa* and *Arabidopsis thaliana*. The phylogenetic tree was generated using Clustal X tool, and the MEGA version 10.6.2 software with default parameters implementing the maximum likelihood (ML) method, the bootstrap test value was set to 1000. The black colored genes are *Medicago sativa LEA_2* Family Genes. The red colored genes are *Arabidopsis thaliana LEA_2* Family Genes. The whole MsLEA_2 family can be divided into two groups: group I and group II.

According to the results of the phylogenetic tree and referring to the results of the *LEA_2* family of *Glycine max* and *Medicago truncatula* (Battaglia and Covarrubias, 2013), both of which are legumes, the *MsLEA_2* family can be further divided into two groups (I, II). Among them, four *AtLEA_2* genes are distributed in group I. It can be speculated that the *MsLEA_2* adjacent to *AtLEA_2* may have a similar function to *AtLEA_2*. For example, *AtLEA14* and 10 alfalfa genes clustered on a separate clade (**Figure 1**). It has been reported that *AtLEA14* can improve the drought or salt stress resistance of plants (Li et al., 2014). The 10 *MsLEA_2* genes, adjacent to *AtLEA14* such as: *MsLEA_2-135*, *MsLEA2*, *MsLEA_2-116* and so on, may have the similar function to improve plants resistance to drought or salt. The related genes in group II are not similar to the *LEA_2* genes in Arabidopsis thaliana. It is speculated that they may be redundant genes provided for evolution or have special molecular functions, but there is no relevant research report yet. It can be seen that the *LEA_2* family has undergone a large degree of differentiation during the long-term evolution.

**Figure 2:**
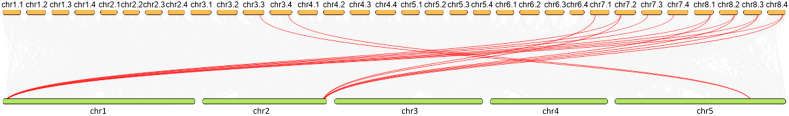
Collinearity analysis of the *MsLEA_2* genes in *Medicago sativa* to *Arabidopsis thaliana.* The gray lines represent collinearity of all genes between the two species; the red line denote collinearity between LEA_2 family members in both species.

To further verify the evolutionary relationship between *AtLEA_2* and *MsLEA_2*, the whole genomes of Arabidopsis thaliana and alfalfa were analyzed using the genome collinearity tool MCScanX (Wang et al., 2013). The results showed that *AtLEA14*, *AtLEA27*, and *AtNHL26* all produced multiple copies in the alfalfa genome (**Figure 2** and **Supplementary Table S2**). No colinearity between *AtLEA26* and any *MsLEA_2* gene was detected. Combined with phylogenetic tree analysis, it was observed that there was a very high sequence similarity between *AtLEA27* and *AtLEA26*. It is suggested that *AtLEA26* was lost in alfalfa evolution, possibly due to functional redundancy.

### Chromosome localization and intraspecific collinearity analysis of the *MsLEA_2* genes

**Figure 3:**
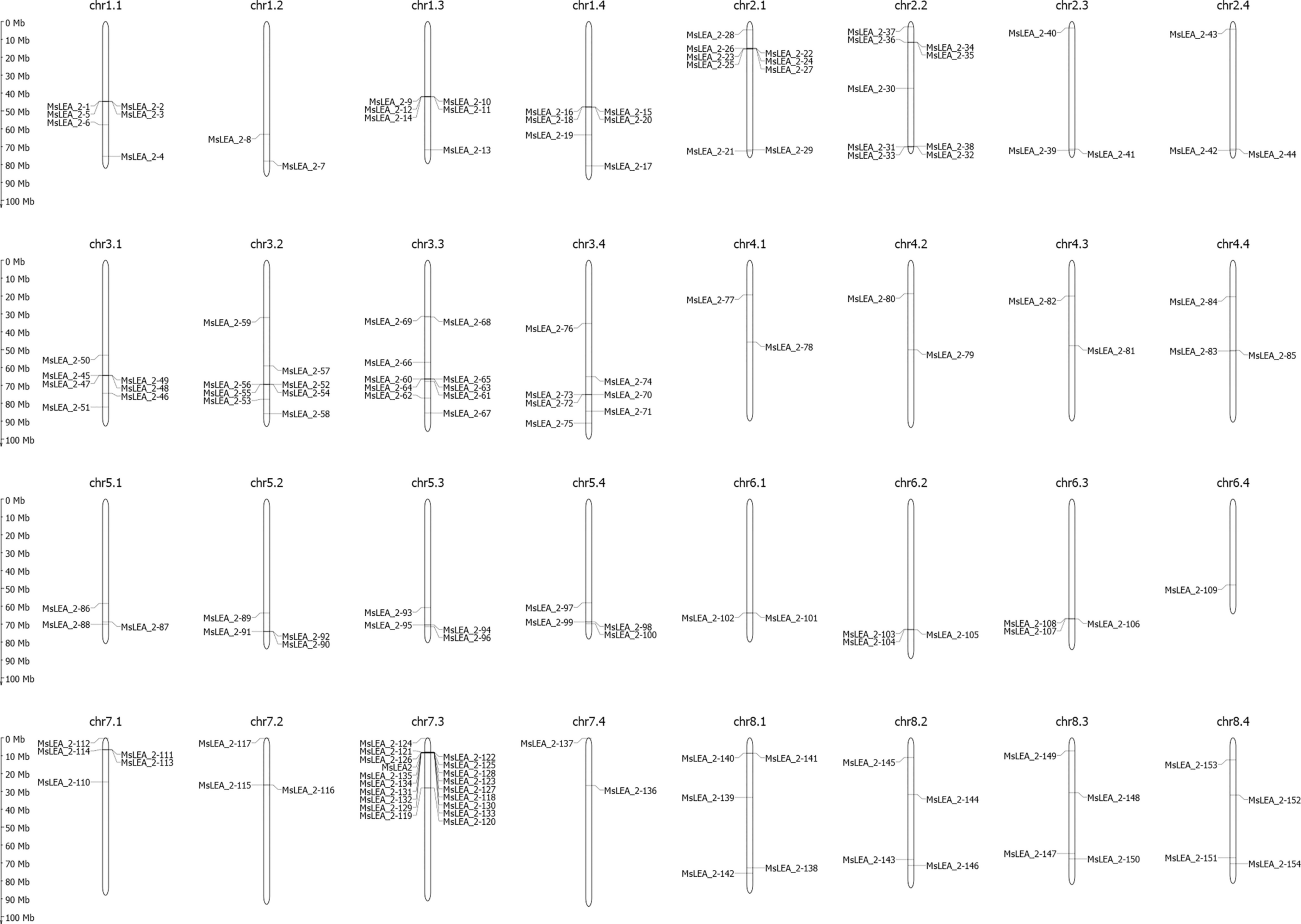
Chromosome distribution of *MsLEA_2* gene family. Chromosome numbers shown at the tops of the chromosome. *MsLEA_2* genes are labeled at the left or right of each chromosome. Scale bars on the left indicate the chromosome lengths (Mb).

**Figure 4:**
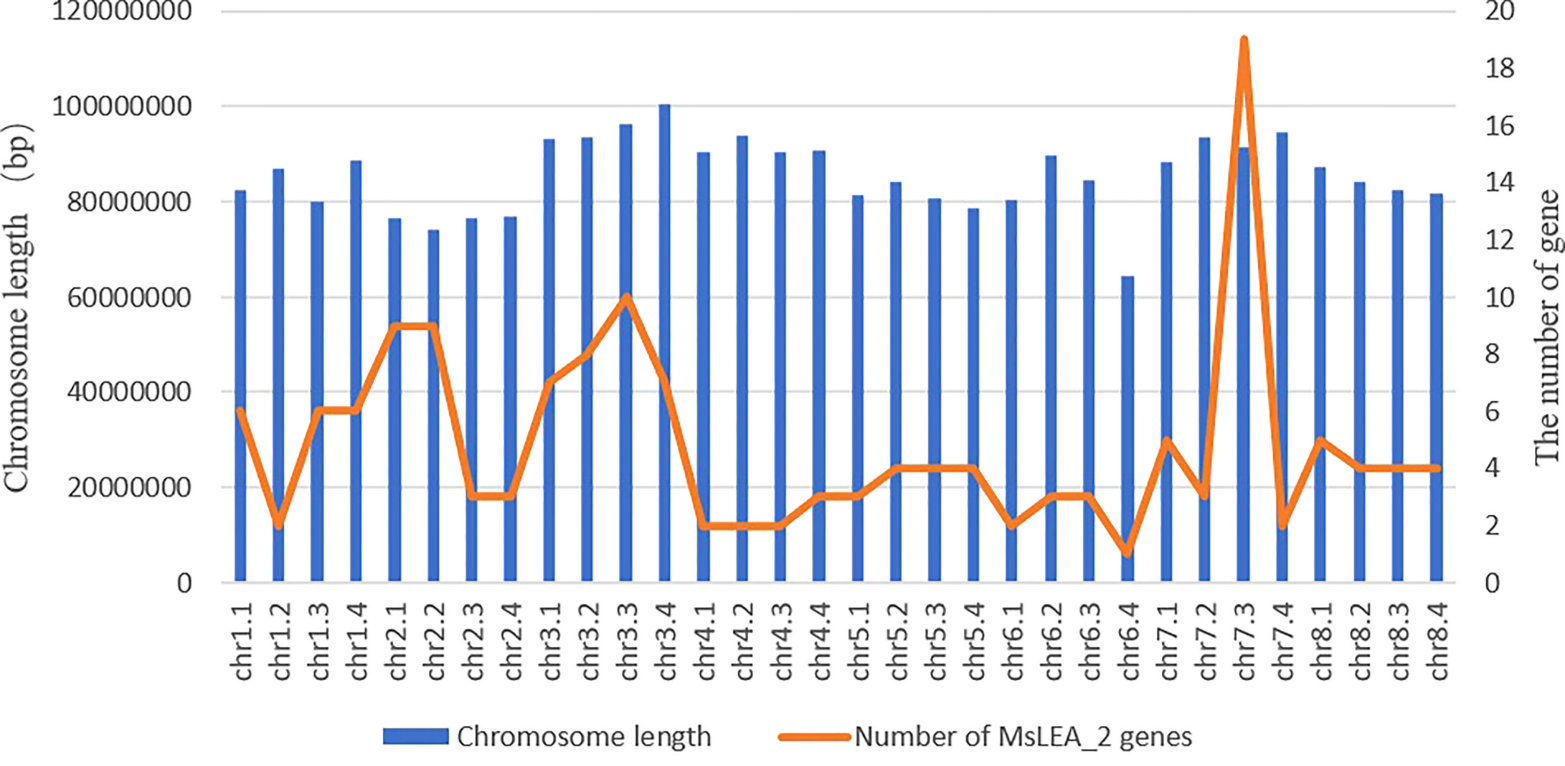
Chromosome length and number of *MsLEA_2* gene family members. The blue columns represent the length of chromosomes, the orange fold line denote the number of *MsLEA_2* genes in every chromosome.

Mapping *LEA_2* on alfalfa chromosome, we found that the *MsLEA_2* genes are unevenly distributed on all 32 chromosomes (**Figure 3**). The third homologous chromosome (Chr7.3) of Chr7 carries the largest number of *MsLEA_2* (19 genes on Chr7.3), while the chromosome Chr6.4 only has only one *MsLEA_2* (*MsLEA_2-109*). And the number of *MsLEA_2* distributed on other chromosomes is range from 2 to 10. This uneven distribution indicates that *MsLEA_2* genes duplication events could have occurred in almost all the chromosomes during alfalfa evolution. There are many *MsLEA_2* genes clustered on the chromosomes. Chromosomes Chr2.1, Chr3.1, Chr3.2, Chr3.3 and Chr7.3 had high-density gene clusters, especially Chr7.3 which carries a highest-density *MsLEA_2* genes cluster containing 16 genes (*MsLEA_2-122*, *MsLEA_2-121*, *MsLEA_2-125*, *MsLEA_2-126*, *MsLEA_2-128*, *MsLEA2*, *MsLEA_2-123*, *MsLEA_2-135*, *MsLEA_2-127*, *MsLEA_2-134*, *MsLEA_2-118*, *MsLEA_2-131*, *MsLEA_2-130*, *MsLEA_2-132*, *MsLEA_2-133*, *MsLEA_2-129*). The number of *MsLEA_2* carried on chr3 (Chr3.1, Chr3.2, Chr3.3, Chr3.4) is the largest (32 genes on Chr3). We found that the number of *MsLEA_2* was not positively correlated with chromosome length (**Figure 4**). In addition, the chromosome distribution map showed that the distribution density of *MsLEA_2* on different chromosomes was different. But when comparing *MsLEA_2* among homologous chromosomes, the distributions of *MsLEA_2* were relatively consistent, only *MsLEA_2-30* in Chr 2.2 has no corresponding gene in other homologous chromosomes of Chr2.

Genes located in or near the telomeric region of chromosomes are easy exchanged during recombination. 14 *MsLEA_2s* (*MsLEA_2-28*, *MsLEA_2-37*, *MsLEA_2-40*, *MsLEA_2-43*, *MsLEA_2-21*, *MsLEA_2-29*, *MsLEA_2-31*, *MsLEA_2-33*, *MsLEA_2-38*, *MsLEA_2-32*, *MsLEA_2-39*, *MsLEA_2-41*, *MsLEA_2-42*, *MsLEA_2-44*) in Chr2 and 4 (*MsLEA_2-112*, *MsLEA_2-117*, *MsLEA_2-124*, *MsLEA_2-137*) in Chr7 located in or near the telomeric regions, suggesting these genes have survived long-term evolution and exchange of alfalfa chromosomes.

**Figure 5:**
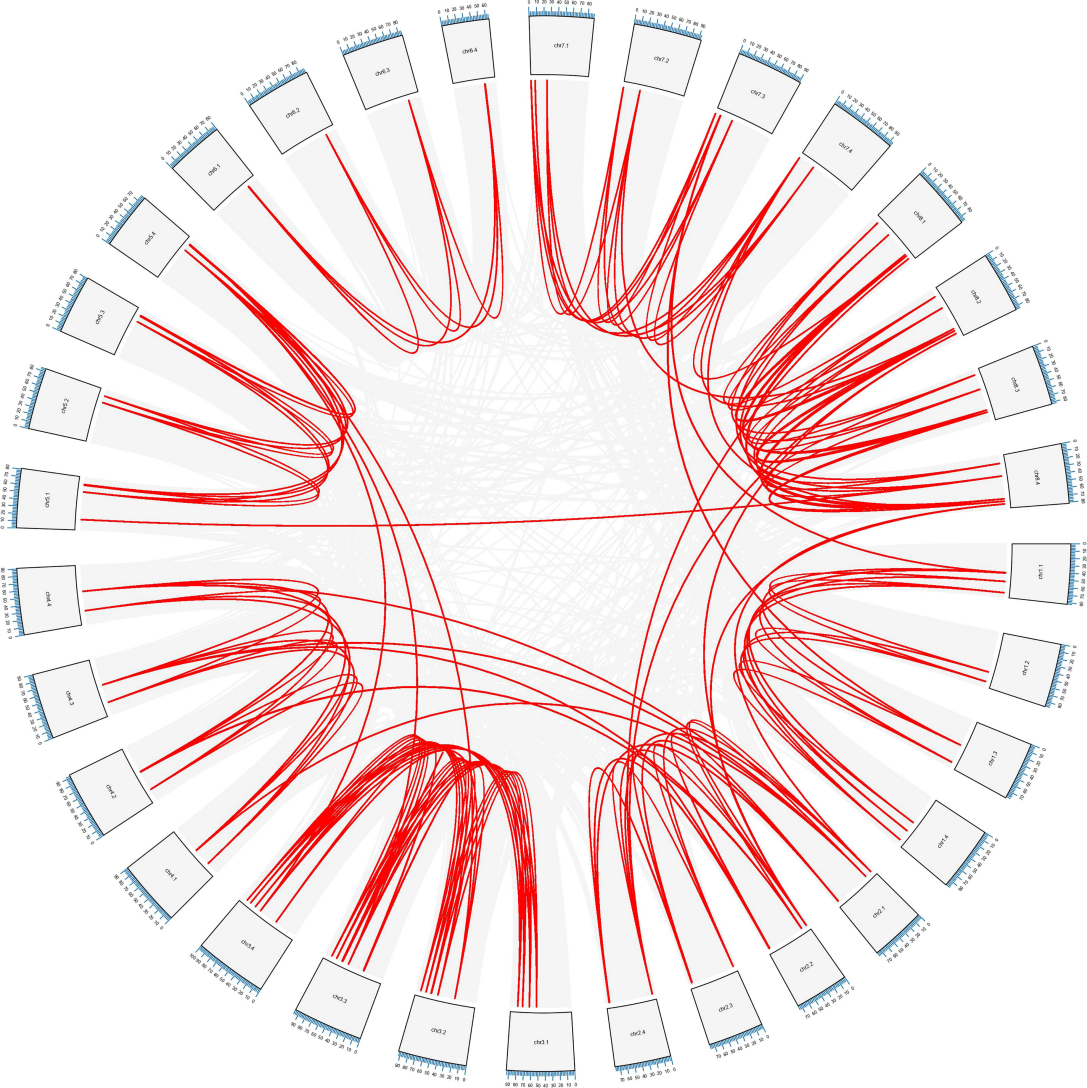
Schematic diagram of the inter-chromosomal relationships of *MsLEA_2* genes. Chromosome numbers are indicated at the outer edge of the circle, while the scale represents megabases (Mb). The lines inside indicate duplicated gene pairs. The red lines represent collinear pairs of the *MsLEA_2* genes, while the gray lines indicate collinear pairs of all alfalfa genes.

The whole alfalfa genome was analyzed using the genome collinearity tool MCScanX (Wang et al., 2013), and all the collinearity and tandem gene duplication in the genome were obtained (**Figure 5**). According to the obtained tandem duplication results, combined with the chromosomal location of *MsLEA_2*, a large number of *MsLEA_2* gene clusters formed by the tandem duplication can be observed, which is consistent with Artur et al., 2019. Among the 155 *MsLEA_2* family members, 85 genes were present in the gene cluster, accounting for 54.83%, and the largest gene cluster was found in Chr7.3, which consisted of 16 genes. There are 10 gene clusters composed of 2 tandem genes, which is the largest number of gene clusters type (**Supplementary Table S3**). According to the results of the collinearity, most of the members of *MsLEA_2* have collinearity between the homologous chromosomes, indicating that the *MsLEA_2* family was formed due to the expansion of genome polyploidization and has strong conservation.

In addition, Chromosome 5.4 (Chr5.4) showed collinear relationship with Chr3.4, Chr3.3, and Chr3.2; Chr5.1 showed collinear relationship with Chr8.4; Chr2.1 showed collinear relationship with Chr4.4, Chr4.3, and Chr4.1; Chr2.2 showed collinear relationship with Chr8.4 and Chr8.2; Chr2.4 showed collinear relationship with Chr8.1 and Chr8.2; Chr7.3 showed collinear relationship with Chr1.3 and Chr1.1. The six pairs of collinearity among these different chromosomes may be due to duplication of chromosome segments.

Among 155 *MsLEA_2* genes, 54.19% have 3 homologous genes, 21.29% have 2 homologous genes, 7.75% have 1 homologous gene, 16.77% have no homologous genes. The phenomenon that genes lost homolog may be caused by retro transposition and evolutionary loss (Xiao et al., 2016).

### Physicochemical properties and subcellular localization of MsLEA_2 protein

The average length of MsLEA_2 proteins is 228 amino acids residues, of which 133 proteins has between 180 and 270 residues (**Table 1**). The longest protein, MsLEA_2-96, contains 324 residues and its corresponding gene is located at Chr5.3. The shortest protein MsLEA_2-114 and MsLEA_2-117, containing 121 residues, their corresponding genes were located at Chr7.1 and Chr7.2 respectively.

The isoelectric point (pI) varies greatly, distributed in the interval of 4.42 (MsLEA_2-116) to 10.46 (MsLEA_2-42), with a mean value of 9.04. Only 14 (9.03%) members had pI<7 and were acidic proteins, and the remaining 141 members (90.96%) had pI>7 and were basic proteins (**Table 1**).

The hydrophilicity values ​​of MsLEA_2 family proteins range from -0.559 (MsLEA_2-146) to 0.284 (MsLEA_2-66), of which only 39 (25.16%) proteins tend to be hydrophobic, and the other 116 (74.48%) proteins tend to be hydrophilic. The predicted subcellular localization indicated that 15 (9.68%) of LEA_2 proteins were located in the cytoplasm, 22 (14.19%) were located outside the cells, four (2.58%) were located in the mitochondria, 28 (18.06%) were located in the nucleus, and the largest number was 86 (55.48%) of LEA_2 proteins located in the cell membrane. For information on sequence ID, gene name, chromosome, number of bases, isoelectric point, relative molecular weight (kD), average hydrophobic index, number of amino acids, and subcellular localization are seen in **Table 1**.

### Analysis of MsLEA_2 and their conserved motifs and domains

**Figure 6:**
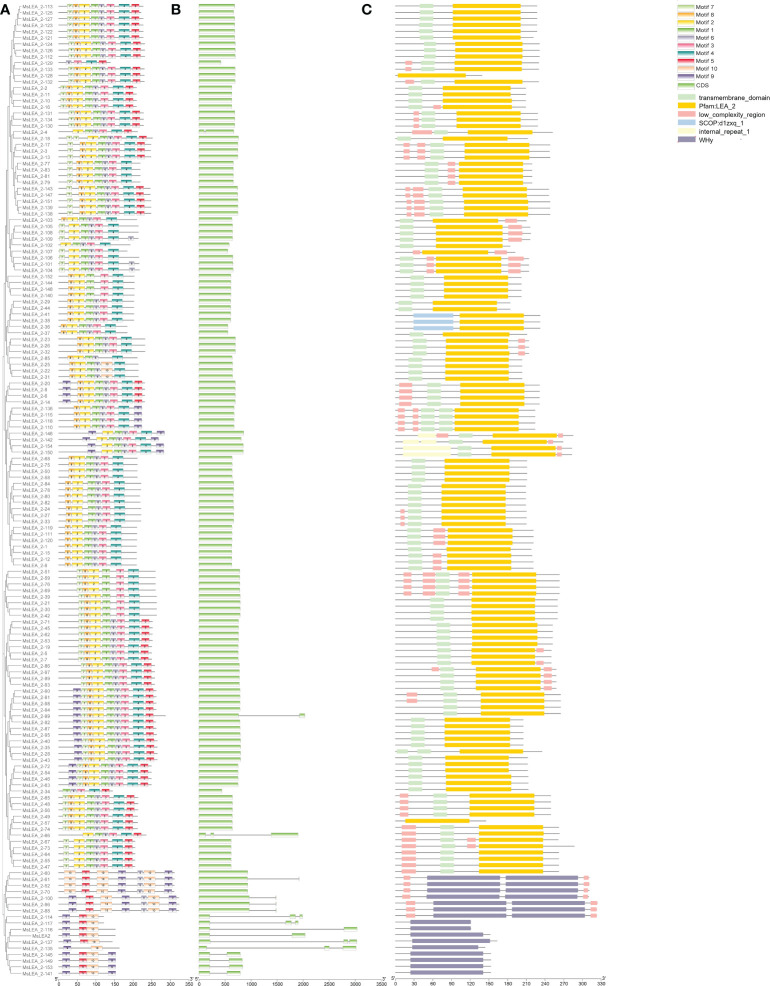
The conservative motifs, gene structure, and conservative domains of *MsLEA _2* gene family. **(A)** The phylogenetic tree constructed by Clustal X tool was shown on the left. Conserved motifs of MsLEA _2 proteins were shown on the right, and these motifs were identified by MEME and boxes with different colors represent different motifs. **(B)** The structure of exons and introns of the *MaLEA* genes were inferred and visualized by TBtools. The genes are arranged according to phylogenetic order. The exons of the genes are colored to be easily distinguishable. **(C)** The conservative domains of MsLEA _2 were predicted by the Pfam database (http://pfam.xfam.org/). The difference conserved domains were marked by difference color boxes, shown on the right.

The conserved motifs of the MsLEA_2 family were identified by MEME software. Among all the MsLEA_2 family proteins, a total of 10 distinct motifs were identified, ranging in length from 15 to 31 residues (**Supplementary Table S4**), and they were unevenly distributed on the MsLEA_2s (**Figure 6**). Each MsLEA_2 contains 2-9 motifs, and none of the motifs appear in all MsLEA_2s. Most of MsLEA_2 contain motif 2 and motif 4. Meanwhile, we also found that MsLEA_2s of the same subgroup in the phylogenetic tree have similar motifs. It is reveals that MsLEA_2s in the same subgroup plays a similar role in plants.

**Figure 7:**
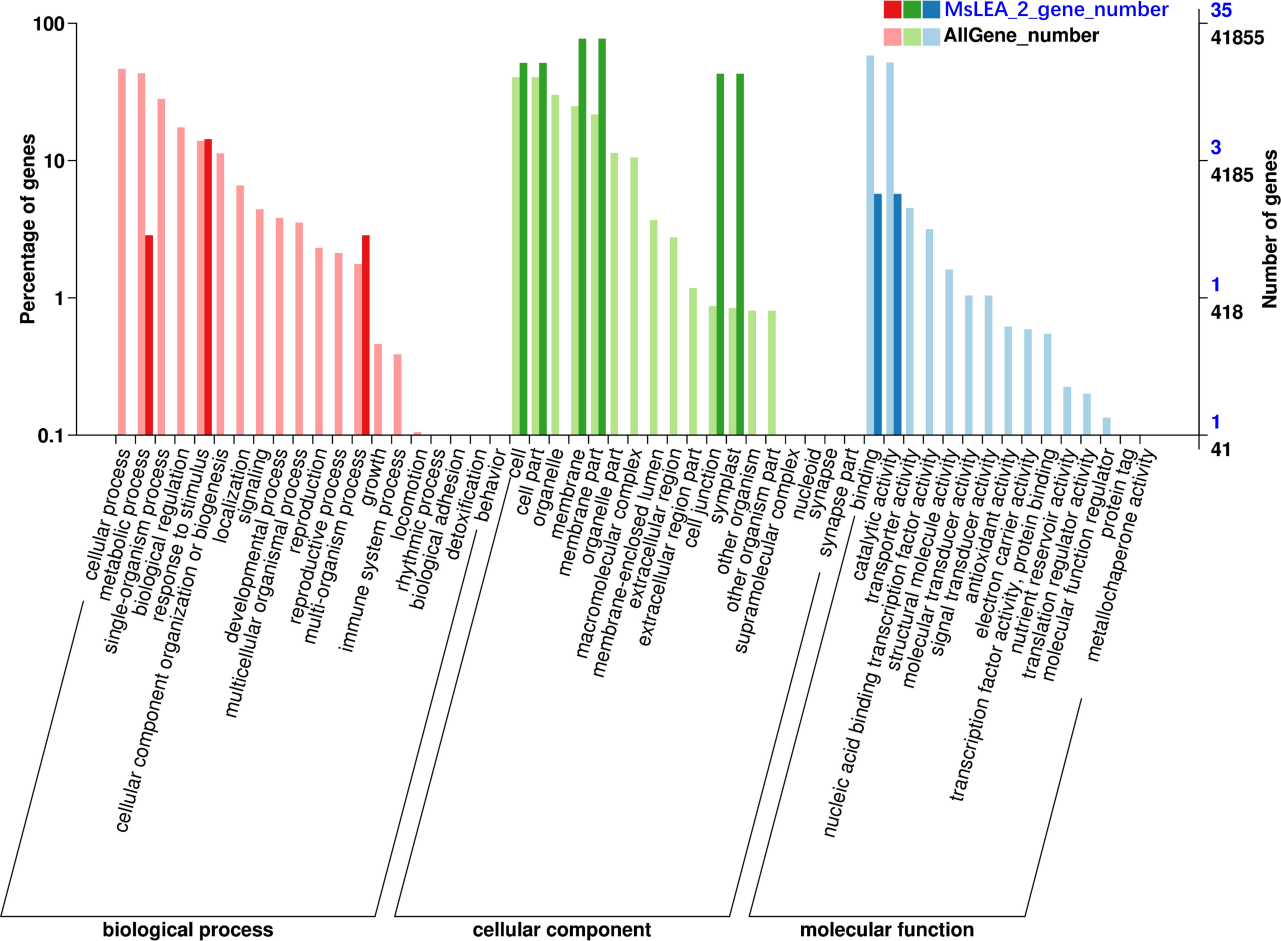
Gene Ontology (GO) classification of MsLEA_2 family genes. The y-axis is the percentage of genes mapped by the term and represents the abundance of the GO term. The x-axis is the definition of the GO terms. The GO function annotation of all genes in alfalfa was used as a control. The MsLEA_2 gene family contain 10 GO categories, which belonged to molecular function (MF), biological process (BP) and cellular component (CC).

Using SMART (http://smart.embl-heidelberg.de/) to further analyze the domains (**Figure 6**), results show that all MsLEA_2 contain LEA_2 (PF03168) or WHy conserved domains, in addition, some members contain receptor spanning transmembrane domains, low complexity regions, and internal repeats. In results, most of the LEA_2 domains were located at the N-terminus of the LEA protein, a few were located at the middle, and there was no LEA_2 domains located at the C-terminus (**Figure 6**). 17 MsLEA_2s contain WHy domains, including 7 genes have two copies of WHy domains. All the MsLEA_2s containing WHy domain are clustered together in the phylogenetic tree. To explore the structural features of the *MsLEA_2* family genes, 155 *MsLEA_2s* were subjected to structural analysis using GSDS 2.0. The results are shown in **Figure 7**. The number of exons in each gene varies from 1 to 3, and the number of introns varies from 0 to 2. Among them, 138 (89.03%) genes had no intron, 12 (7.74%) contained one intron, 5 (3.32%) contained two introns.

### GO functional annotation of *MsLEA_2*

The 155 *MsLEA_2* genes obtained were functionally annotated using Blast2GO. It is showed that the *MsLEA_2* gene family contained 10 GO categories, which belonged to molecular function (MF), biological process (BP) and cellular component (CC) (**Figure 7**). Taking the GO function annotation of all genes in alfalfa as a control, it can be observed that in the molecular function (MF) category, the *MsLEA_2* family is mainly enriched in binding function (50%) and catalytic function (50%); in biological process (BP) category, the MsLEA_2 was mainly in response to stimulation (71.42%) and polyorganic processes (14.29%); in the cellular component (CC) category, MsLEA_2 was mainly in cell membrane (45%), cell junctions (25%) and symplasts (25%), this is consistent with the predicted subcellular localization results.

### Analysis of *cis*-elements in the promoter region of *MsLEA_2*

**Figure 8:**
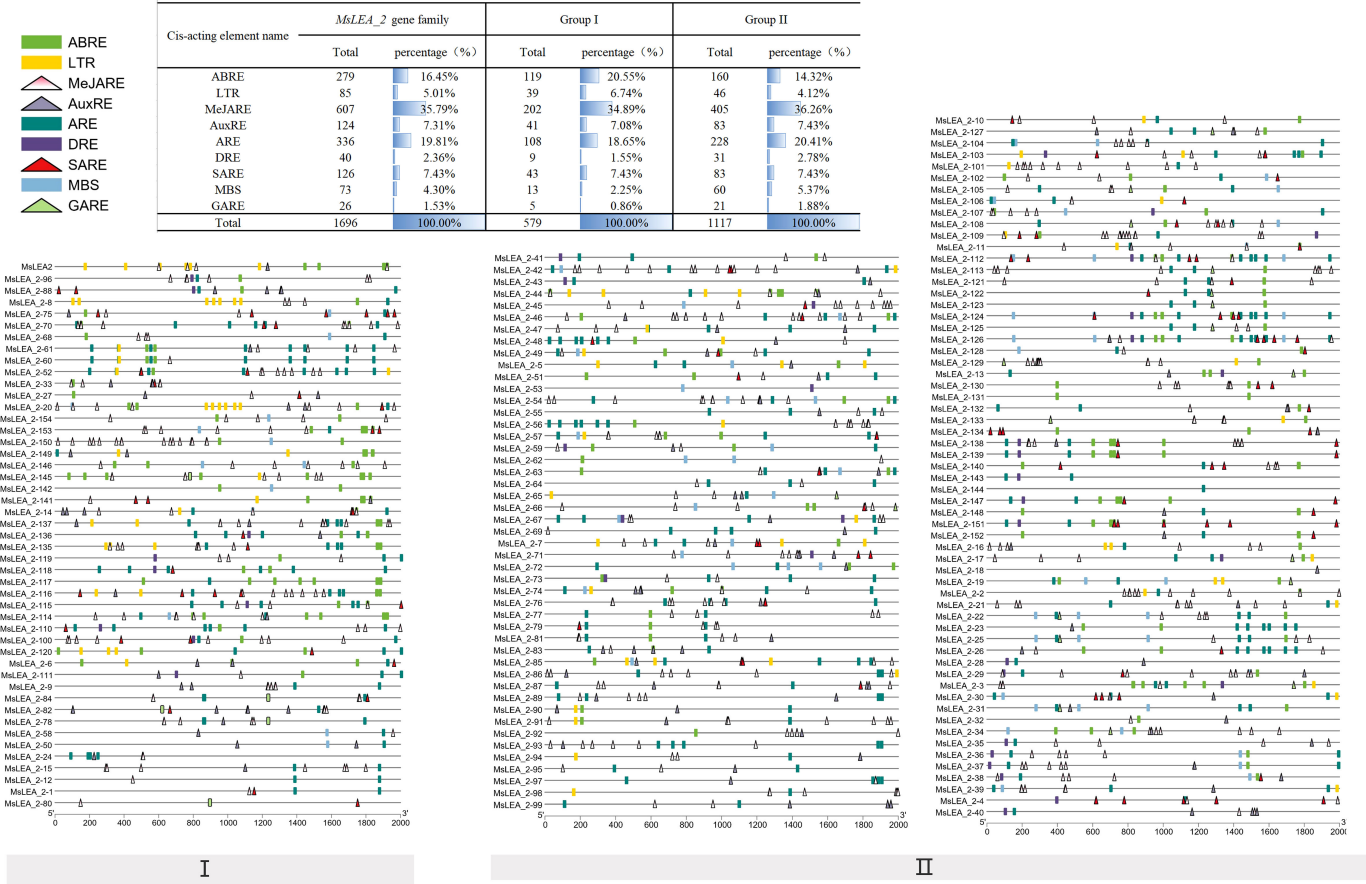
Predicted *cis*-element analysis of the *MsLEA_2* gene family. Different colors and shapes represent different *cis*-elements. Number of each *cis*-element of the *MsLEA_2* gene promoter region (2.0 kb upstream of the translation start site) was shown in the table on the top of the figure. Abbreviations were used to indicate different *cis*-elements: “ABRE” refer to Abscisic Acid Responsive Element; “MeJARE” refer to Methyl Jasmonate Responsive Element; “DRE” refer to Drought Response Element; “MYB” refer to MYB Transcription Factors; “MYC” refer to MYC Transcription Factors.

The 2kb upstream of the initiation codon (ATG) of 155 *MsLEA_2* genes were analyzed online using Plant CARE (http://bioinformatics.psb.ugent.be/webtools/plantcare/html/) (Lescot et al., 2002). It is found that the promoter of 155 *MsLEA_2* included a total of 31998 *cis*-elements. Among them, the elements related to the hormone signaling pathway include 770 methyl jasmonate response elements (MeJAREs), 413 abscisic acid response elements (ABREs), 208 salicylic acid response elements (SAREs), 162 auxin response elements (AuxREs), and 157 gibberellin response elements (GAREs). Elements related to abiotic stress include 490 anaerobic inducible response elements (AREs), 154 low temperature response elements (LTRs), 133 drought stress response elements (DREs), 90 defensive stress response elements, 5 wound response elements and so on. In the *MsLEA_2* family, each member contained on average 2.665 ABREs, 0.9613 MYB binding sit (MBS), 0.9419 LTRs, and only 6.451% of the members did not contain the above three corresponding elements (**Figure 8**). These results indicate that the members of *MsLEA_2* may be stimulated under abiotic stress, and play their role of protecting plants.

### Expression analysis of *MsLEA_2* under Al stress

**Figure 9:**
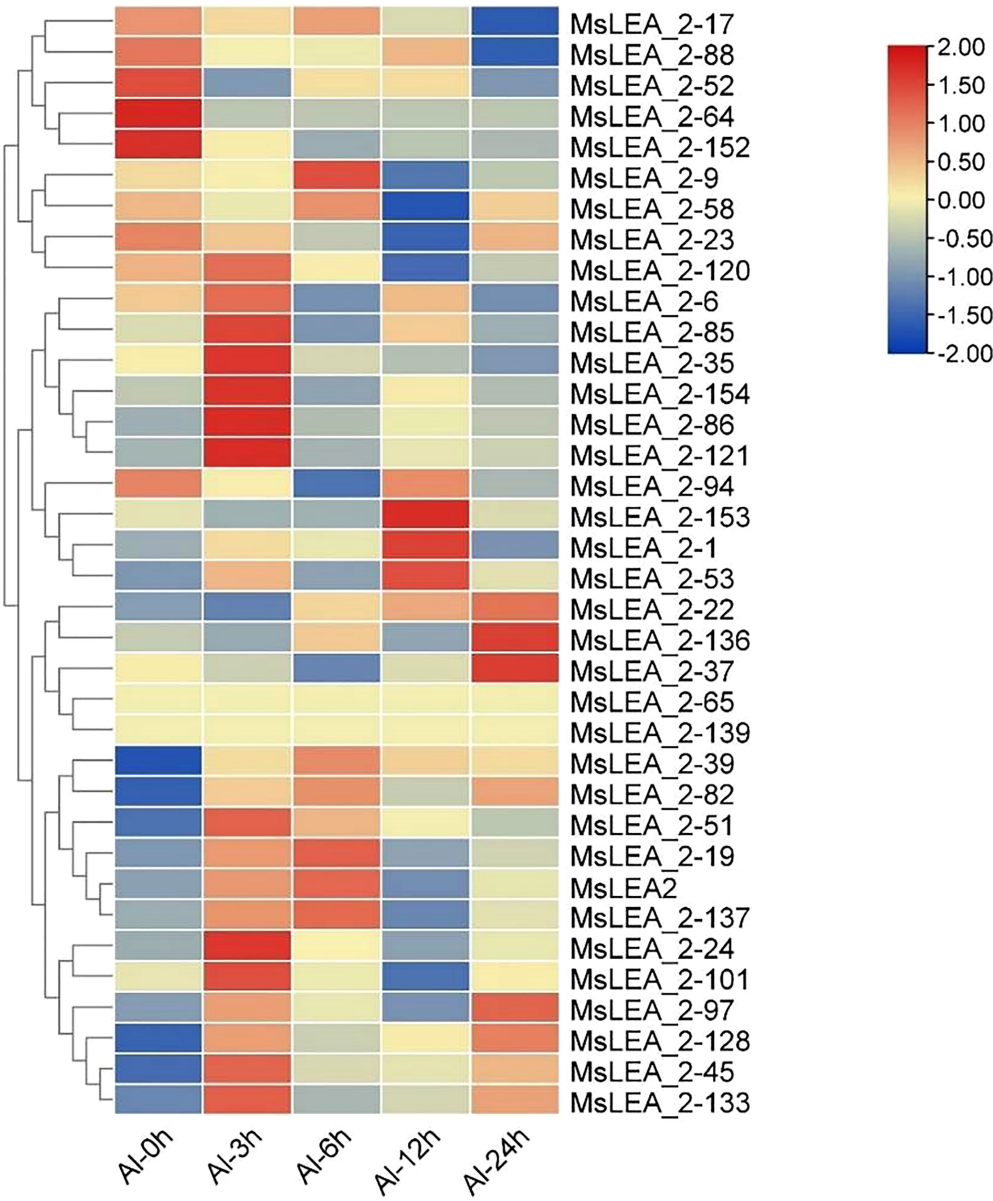
RNA-seq expression profiles of parts of *MsLEA_2* genes under Al treatment. The heatmap was constructed using TBtools. The color scale on the right represents relative expression levels: Red represents high level and blue represents low level.

**Figure 10:**
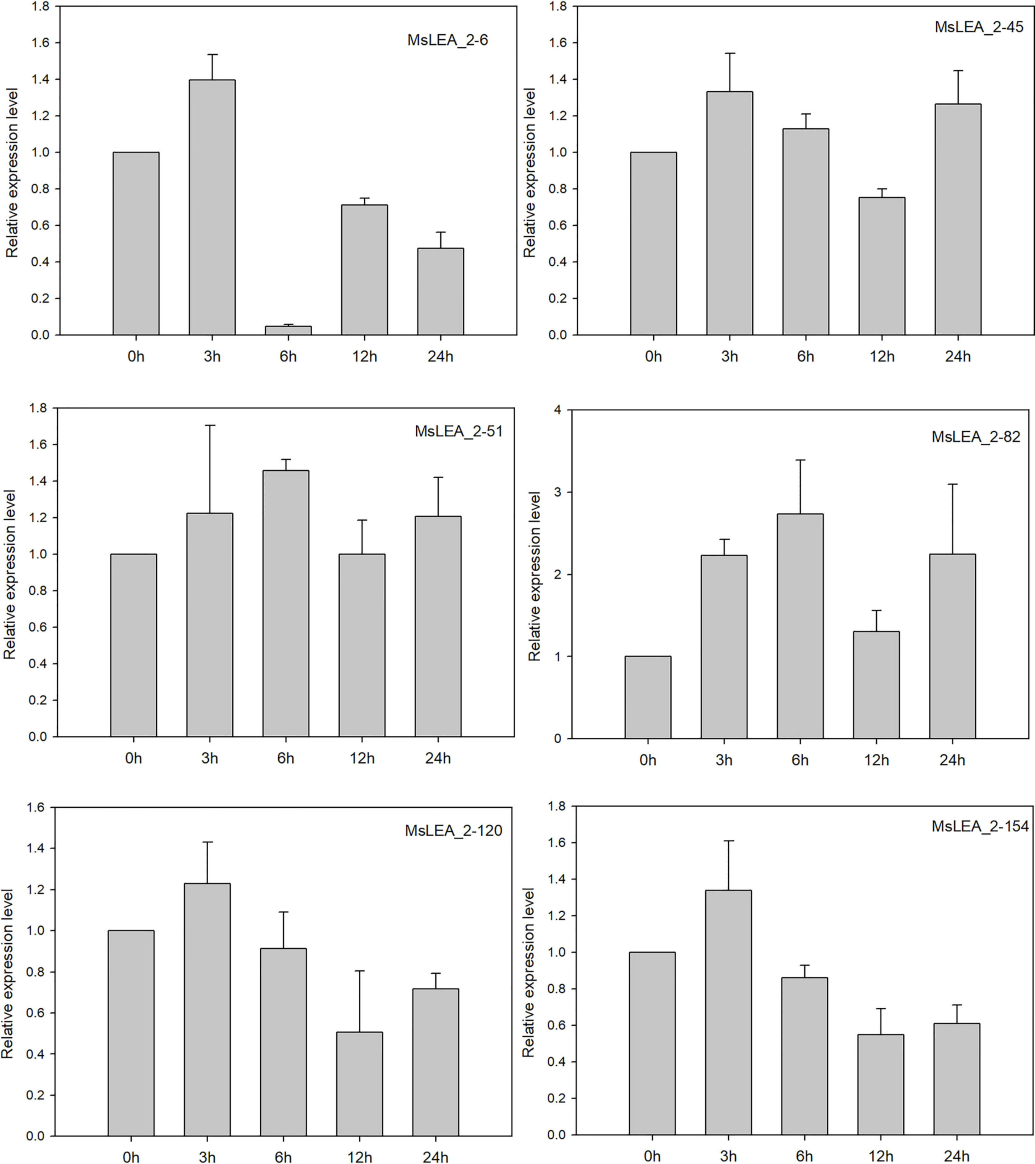
The relative expression levels of six *MsLEA_2* genes under Al stress were analyzed by qRT-PCR. 14-days-old alfalfa seedlings were sampled after 0 h, 3 h, 6 h, 9 h, 12 h, and 24 h under 100μM Al3+ treatment. The relative expression of six *MsLEA_2* genes (*MsLEA_2-6*, *MsLEA_2-45*, *MsLEA_2-51*, *MsLEA_2-82*, *MsLEA_2-120*, *MsLEA_2-154*) were calculated using the 2 ^-△△CT^ method with housekeeping gene MsEF-a as an endogenous control.

To analyzing the expression profiles of alfalfa *MsLEA_2* genes in response to abiotic stress, we analyzed the expression of 36 *MsLEA_2* genes from previous RNA-seq data in alfalfa in response to Al stress. The expression of *MsLEA_2* was down-regulated (accounting for 14, 41.67% of the total) after 100 μM Al^3+^ stress treatment, while the expression of 2 *MsLEA_2* remained unchanged (5.56%), and the expression 20 of 36 *MsLEA_2* genes were up-regulated (55.56%) (**Figure 9**). The expression of six genes (*MsLEA_2-6*, *MsLEA_2-45*, *MsLEA_2-51*, *MsLEA_2-82*, *MsLEA_2-120*, *MsLEA_2-154*) under Al stress were also analyzed by qRT-PCR. The results showed that all six genes were up-regulated when alfalfa exposed to Al stress 3 or 6 hours (**Figure 10**).

## Discussion

LEA_2 is an important resistance protein in plants, and is very sensitive to water stress (Jaspard and Hunault, 2014). With the completion of the sequencing of various plant genomes, a comprehensive analysis of the evolution and function of different plant gene families has become possible. At present, the structure and distribution of *LEA_2* gene family in the genome of some plants have been identified and analyzed, such as *Arabidopsis thaliana* (Dang et al., 2014), *Oryza sativa* (Xue et al., 2021), *Zea mays* (Li and Cao, 2015), etc. However, the genome-wide level of alfalfa *LEA_2* family genes analysis has not yet been reported. In this study, the alfalfa *LEA_2* family members (*MsLEA_2s*) were identified through the bioinformatics study, and *MsLEA_2s’* genetic evolution, physicochemical properties and expression patterns under abiotic stress were analyzed. The results indicated that the *MsLEA_2* had the potential to resist stress under abiotic stress.

Referring to the genome sequencing data of alfalfa (Xinjiang Daye), the Arabidopsis *LEA_2* genes and the PFAM numbers of LEA_2 (PF03168) (Finn et al., 2014), 155 *LEA_2* gene family members in alfalfa have been identified. At present, a number of *LEA_2* genes have been founded in many species. For example, four *LEA_2* genes were found in *Arabidopsis thaliana*, 64 were found in *Oryza sativa*, 157 in cotton (*Gossypium. hirsutum*), 56 in rye (*Secale cereale*) and 71 in *Medicago truncatula* (Galau et al., 1986). Artur et al., 2019 analyzed 60 fully sequenced genomes and found that there are 3126 members in *LEA_2* gene, and confirmed that *LEA_2* is the largest subfamily of the *LEA*. We had found 155 *LEA_2* genes in alfalfa, which is one of the largest *LEA_2* families as we known, second only to 157 in cotton (*Gossypium. hirsutum*). 155 alfalfa *LEA_2* genes were subjected to multiple sequence alignment analysis and a phylogenetic tree containing 159 LEA_2 proteins including the whole of alfalfa and Arabidopsis LEA_2 proteins was generated (**Figure 1**). *MsLEA_2* genes can be divided into two groups, interestingly only Group I is adjacent to *AtLEA_2*, suggesting Group II may be redundant bases for evolution, or Group II has other special molecular function. In RNA-Seq analysis, we found 59.09% of Group II were up-regulated under stress, revealing some of Group II are also respond to stress.

Alfalfa is a self-incompatibility cross-pollinated tetraploid plant (number of chromosomes: 2n = 4 × = 32), in which the bivalent pairing is random and non-preferential, resulting in a very complex genome, so the intuitive chromosomal location of the *MsLEA_2* gene is necessary. We found that the *MsLEA_2* genes are unevenly distributed on all 32 chromosomes, like *LEAs* observed in other species (Altunoglu et al., 2017; Ibrahime et al., 2019; Piyatissa and Bandupriya, 2021). This phenomenon may be due to the fact that the *LEA_2s* or other *LEAs*, which are widely distributed on multiple chromosomes, can produce enough resistance proteins to cope with the effects of stress on plants. And this arrangement may be beneficial for triggering the whole genome responses under stress (Piyatissa and Bandupriya, 2021). Many *MsLEA_2* genes clusters are founded on several chromosomes, and the high-density gene clusters was founded on the chromosomes such as: Chr2.1, Chr7.3 and so on. It is expected that these chromosomes regions with high-density gene cluster may contribute to the expression of LEA_2 proteins under stress conditions (Piyatissa and Bandupriya, 2021). Chromosome Chr7.3 carries the largest number of *MsLEA_2s*, and it contains the highest-density *MsLEA_2* gene cluster. And *MsLEA_2-124* located to the telomeric regions of Chr7.3. The unique structure of *MsLEA_2* distribution on Chr7.3 reveals the special role of Chr7.3 in ensuring the function of *MsLEA_2*.

Gene duplication is a major mechanism for increasing genetic complexity and diversity, which cause the emergence of new genes and plays an important role in genomic evolution (Moore and Purugganan, 2003; Zhang et al., 2022). The modes of gene duplication include whole genome duplication or polyploidization, tandem duplication, segmental duplication and retro-transposition (Kahn and Raphael, 2008; Xiao et al., 2016; Jain et al., 2017; Van De Peer et al., 2017). Based on the obtained tandem repeat results, combined with the chromosomal location of the members of the *MsLEA_2s*, we found that among the 155 members of the *MsLEA_2s*, 54.83% existed in gene clusters formed by tandem repeats, and the largest gene cluster existed on Chr7.3, which consisted of 16 tandem repeat genes (**Figure 3**). Collinearity analysis intuitively demonstrated the way of *MsLEA_2* family members expanding through duplication (**Figure 5**). Most of the *MsLEA_2* members have collinearity between homologous chromosomes, the *MsLEA_2s* with collinearity showed highly conserved, indicating that the *MsLEA_2* family was formed due to the expansion of genome polyploidization (**Figure 5**). Only a few pairs of *MsLEA_2s* on non-homologous chromosomes have collinearity, which may be caused by segmental duplication. All the results supported the hypothesis that the primary mode of gene duplication of *LEA_2s*, an atypical LEA family, is tandem duplication (Artur et al., 2019). Tandem duplication drives *LEA_2s* to expand and diversify, and may lead *LEA_2s* functional diversification (Artur et al., 2019).

According to gene structure analysis, most of the *MsLEA_2* genes had no introns while only 10.97% harbor one or two introns (**Figure 6**). The structural characteristics of *MsLEA_2s* are consistent with that of the functional genes in response to abiotic and biotic stress. In many species, it was found that genes with major functions on abiotic and biotic stress factors always had few introns (Lan et al., 2013; Magwanga et al., 2018; Piyatissa and Bandupriya, 2021). The presence of introns in the genome is considered to place a burden on the host, because introns need to be deleted by a spliceosome which is one of the largest molecular complexes in cells and consists of five small nuclear RNAs and many proteins (Wahl et al., 2009). The study also found that transcription of intron consumed additional time and energy (Lane and Martin, 2010), introns can prolong the length of newborn transcripts, and result in additional transcription costs (Jeffares et al., 2008). In stress resistant genes, the loss of intron can save time and improve transcription efficiency (Jeffares et al., 2008).

The protein structure domain analysis was showed that MsLEA_2 proteins all contain LEA_2 or WHy conserved domains (**Figure 6**). The common domain of LEA_2 family is LEA_2 domain. The main characteristic of LEA_2 domain is that they are natively folded and more hydrophobic than other LEA proteins (Hundertmark and Hincha, 2008; Jaspard et al., 2012). The WHy domain is the core domain of a non-specific binding site in the LEA_2 gene family. Numerous studies have shown that the WHy-containing gene is a water stress-related gene (Jaspard and Hunault, 2014). *In vitro* experiments proved that WHy domain has the function of protecting protein from denaturation (Jaspard and Hunault, 2014). In addition, protein transmembrane regions, low-complexity regions, and internal repeats are also present. Combined with the distribution of gene clusters on chromosomes, we found that *MsLEA2* and *MsLEA_2-135* in the high-density *MsLEA_2* gene cluster of Chr 7.3, and *MsLEA_2-60* in gene cluster of Chr 3.3 contain WHy domains, hinting that they have important roles in stress tolerance and may have similar mechanism. The transmembrane region of receptor has the property of spanning the phospholipid bilayer of the cell membrane and can penetrate the membrane permeability barrier, so it plays a key role in many important cellular physiological processes, including signal and energy conversion, active transport, ion flow and nerve conduction, etc. Low-complexity regions are ubiquitous regions in proteins, and some literature speculates that such regions may lead to poor crystallization of proteins. Like leucine zippers, many of low-complexity regions have important biological functions (Sharpe et al., 2010). According to the GO functional annotation, MsLEA_2 protein was mainly enriched in cell membrane, cell junction and symplast, and was generally distributed in cells (**Figure 7**). Predicted subcellular localization and GO-CC annotation indicated that MsLEA_2 protein was mainly enriched in the cell membrane. It has been reported that members of the *LEA* gene family of Arabidopsis thaliana are widely distributed in multiple organelles of plants (Candat et al., 2014). Based on the analysis results of this study, it can be proposed that the LEA protein is on the organelle membrane in response to the mechanism hypothesis of aluminum (Al) stress in some way. The results will be verified in subsequent experiments by constructing a subcellular localization vector and *in vitro* affinity.

**Figure 11:**
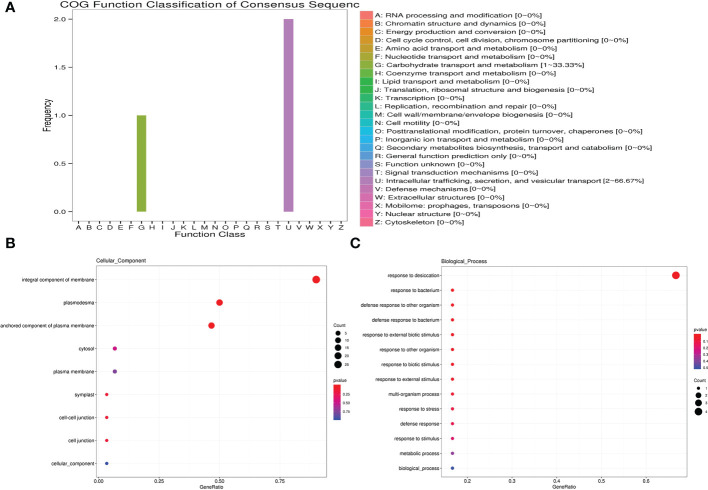
COG, GO-BP, and GO-CC enrichment analysis of up-regulated *MsLEA_2* genes. **(A)** Cluster of Orthologous Groups of proteins (COG) analysis of up-regulated *MsLEA_2* genes. **(B)** GO Cellular Component (GO-CC) analysis of up-regulated *MsLEA_2* genes. **(C)** GO Biological Process (GO-BP) analysis of up-regulated *MsLEA_2* genes.

ABRE, MBS and LTR *cis*-elements are widely distributed in the promoter regions of *MsLEA_2* genes (**Figure 8**). These *cis*-elements are involved in the regulation of downstream gene responses under abiotic stress (Bartels and Sunkar, 2005). The widely distribution of ABRE, MBS and LTR *cis*-elements suggested that *MsLEA_2*s may responses to abiotic stress. According to previous research, spraying low concentration (5 μmol/L) of jasmonic acid can significantly alleviate the damage of Al stress on alfalfa seedlings (Li and Cao, 2015). In this study, we found that there are abundant MeJAREs (methyl jasmonate response elements) in the promoter region of *MsLEA_2* genes, suggesting that the *MsLEA_2*s are regulated by jasmonic acid. We hypothesized that *MsLEA_2* responds to Al stress. So, the expression of *MsLEA_2* gene under Al stress was further analyzed, and the results showed that most *MsLEA_2* genes were up-regulated under Al stress (**Figures 9** and **10**), and the up-regulated *MsLEA_2* gene COG annotations were enriched in “carbohydrate transport and metabolism” and “intracellular transport, Secretion and Vesicle Transport” (**Figure 11**). It indicated that members of the *MsLEA_2* family could respond to abiotic stress responses of plants (Artur et al., 2019). They are likely to improve plant resistance to abiotic stress by participating in plant carbohydrate transport or metabolism.

## Conclusion

In this study, we have identified 155 *LEA_2* family members in alfalfa. The *MsLEA_2*s are distributed on all 32 chromosomes. Among them, 54.83% genes were present in the gene clusters, and the chromosome Chr7.3 carries the largest number of *MsLEA_2* (19 genes). Chr7.3 has a unique structure of *MsLEA_2* distribution, which reveals a possible special role of Chr7.3 in ensuring the function of *MsLEA_2*. Transcriptional structure analysis revealed that the number of exons in each gene varies from 1 to 3, and introns varies from 0 to 2. *Cis*-element analysis identified that the promoter regions of *MsLEA_2* are rich in ABRE, MBS, LTR, MeJARE, indicating *MsLEA_2* genes have stress resistance potential under abiotic stress. Depending on previously RNA-seq, our analysis the expression of most *MsLEA_2* members was up-regulated under Al stress, which were further confirmed by qRT-PCR. The results of this study manifested novel insights into phylogenetic relationships and possible functions of alfalfa *LEA_ 2*s. And the findings will be helpful for the future functional analysis of the LEA_ 2 proteins family.

## Data availability statement

The datasets presented in this study can be found in online repositories. The names of the repository/repositories and accession number(s) can be found in the article/**Supplementary Material**.

## Author contributions

PZ and YZ: designed experiment and completed the manuscript. YZ and NF: completed experiments and major bioinformatics analysis. WW, SL, and XM: prepared materials and reagents and experimental methods. PZ and YA discussed the whole data and finished the revision. All authors contributed to the article and approved the submitted version.

## Funding

This work was supported by the National Natural Science Foundation of China (Nos. 31872419, 31872408, and 32201447) and National Project on Sci-Tec Foundation Resources Survey (No. 2017FY100600).

## Conflict of interest

The authors declare that the research was conducted in the absence of any commercial or financial relationships that could be construed as a potential conflict of interest.

## Publisher’s note

All claims expressed in this article are solely those of the authors and do not necessarily represent those of their affiliated organizations, or those of the publisher, the editors and the reviewers. Any product that may be evaluated in this article, or claim that may be made by its manufacturer, is not guaranteed or endorsed by the publisher.
