# Supplementary material for: Genome−wide identification and analysis of LEA_2 gene family in alfalfa (Medicago sativa L.) under aluminum stress
Source: Front Plant Sci. 2022 Nov 28;13:976160. doi: 10.3389/fpls.2022.976160 (PMC9742422; doi:10.3389/fpls.2022.976160)
Supplement: Supplementary file 1 [file DataSheet_1.docx]

**Supplementary Files**

**Supplementary Table S1. Primer sequences used for qRT-PCR**

| **Seq.ID** | **Gene name** | Forward sequence | Reverse sequence |
| --- | --- | --- | --- |
| MS.gene49981.t1 | *MsLEA_2-6* | 5‘-ATGTTATCGGGTCGATGCAG-3’ | 5‘-AACATCACCGTACCATCAGC-3’ |
| MS.gene008386.t1 | *MsLEA_2-45* | 5‘-CAGTCCTTCACATCCACCTC-3’ | 5‘-CTGTCTACGGAGTATTTGGGAA-3’ |
| MS.gene32816.t1 | *MsLEA_2-51* | 5’-CCATCCTCACCAACCACAAT-3‘ | 5’-CGGAGAAGGCAGTAACAGTG-3‘ |
| MS.gene66696.t1 | *MsLEA_2-82* | 5’-ACGCTAAGCAATCTTCAGCA-3‘ | 5’-CGGAGATCCAACTTCCAACC-3‘ |
| MS.gene007136.t1 | *MsLEA_2-120* | 5’-TACAGAACCGCCATACCTCC-3‘ | 5’-AACCTTCCACCGAACCCTAC-3‘ |
| MS.gene067336.t1 | *MsLEA_2-154* | 5’-ATCACAGTCACAAGTGCTGC-3‘ | 5’-GAGGCGTAATCGGATGGTTT-3‘ |

Supplementary Table S2. Interspecific collinearity analysis of *Medicago sativa* and *arabidopsis thaliana*

| Chromosome number and LEA_2 gene name of *arabidopsis thaliana* | | Chromosome number and gene name corresponding to *Medicago sativa* | | | |
| --- | --- | --- | --- | --- | --- |
| Chr1 | | *AtLEA14* | | chr7.2 | *MsLEA_2-116* |
|  |  |  |  | chr7.3 | *MsLEA2* |
|  |  |  |  | chr7.4 | *MsLEA_2-137* |
|  |  |  |  | chr8.1 | *MsLEA_2-141* |
|  |  |  |  | chr8.2 | *MsLEA_2-145* |
|  |  |  |  | chr8.3 | *MsLEA_2-149* |
|  |  |  |  | chr8.4 | *MsLEA_2-153* |
| Chr2 | | *AtLEA27* | | chr7.1 | *MsLEA_2-114* |
|  |  |  |  | chr7.2 | *MsLEA_2-116* |
|  |  |  |  | chr8.1 | *MsLEA_2-141* |
|  |  |  |  | chr8.2 | *MsLEA_2-145* |
|  |  |  |  | chr8.3 | *MsLEA_2-149* |
|  |  |  |  | chr8.4 | *MsLEA_2-153* |
| Chr5 | | *AtNHL26* | | chr3.3 | *MsLEA_2-68* |
|  |  |  |  | chr3.4 | *MsLEA_2-75* |

Supplementary Table S3. Analysis of gene clusters formed by members of *MsLEA _ 2* family

| Number of tandem genes in a single gene cluster | 2 | 3 | 4 | 5 | 6 | 16 |
| --- | --- | --- | --- | --- | --- | --- |
| Number of gene clusters | 10 | 6 | 5 | 1 | 1 | 1 |
| Percentage in MsLEA_2 Family （%） | 12.90 | 11.61 | 12.90 | 3.23 | 3.87 | 10.32 |

Supplementary Table S4. Motif Information Table of *MsLEA _ 2* Gene Family Protein

| **NO.** | **Motif sequence** | **Width** |
| --- | --- | --- |
| 1 | YNFDVTVTARNPNKKIGIYYD | 21 |
| 2 | LIFWLIVRPKAPKFTVTDATJTQFNLTSP | 29 |
| 3 | LPPFYQGHKNTTVLSPVLKGQ | 21 |
| 4 | LNKDKNKGVVGJDVKLRARVRFKVGSFKS | 29 |
| 5 | KVKCDLKVDLLSANAKIVSGN | 21 |
| 6 | DIEAEAFYKDQRLCN | 15 |
| 7 | RRKHRRRCCCCCLCW | 15 |
| 8 | FIKJILILIVJLGJA | 15 |
| 9 | KJPKEKVGEVPKPEAARRDVD | 21 |
| 10 | IPDPGSIKAHGTTTVDVPVTVPYDDIKSTYA | 31 |
